# Platelet-released polyphosphate controls thrombin generation

**DOI:** 10.1016/j.rpth.2026.106953

**Published:** 2026-09-23

**Authors:** Jack M. Kurth, Xu Zhang, Joseph E. Aslan, Owen J.T. McCarty

**Affiliations:** 1 Department of Biomedical Engineering, Oregon Health & Science University, Portland, Oregon, USA; 2 Knight Cardiovascular Institute, Oregon Health & Science University, Portland, Oregon, USA

The rate of thrombus formation is mechanistically propagated by the initiation, catalysis, and inhibition of thrombin generation (Figure). At a site of vascular disease or injury, the kinetics of thrombin generation are dependent on the transport of blood cells and coagulation factors to a catalytic bed of aggregating platelets [1]. Thrombogenesis is initiated by extracellular matrix proteins such as collagen in concert with cell-surface glycoproteins including tissue factor (TF), a major cofactor of activated factor (F)VII (FVIIa), which collectively recruits and activates platelets to subsequently catalyze thrombin generation [2].

**Figure:**
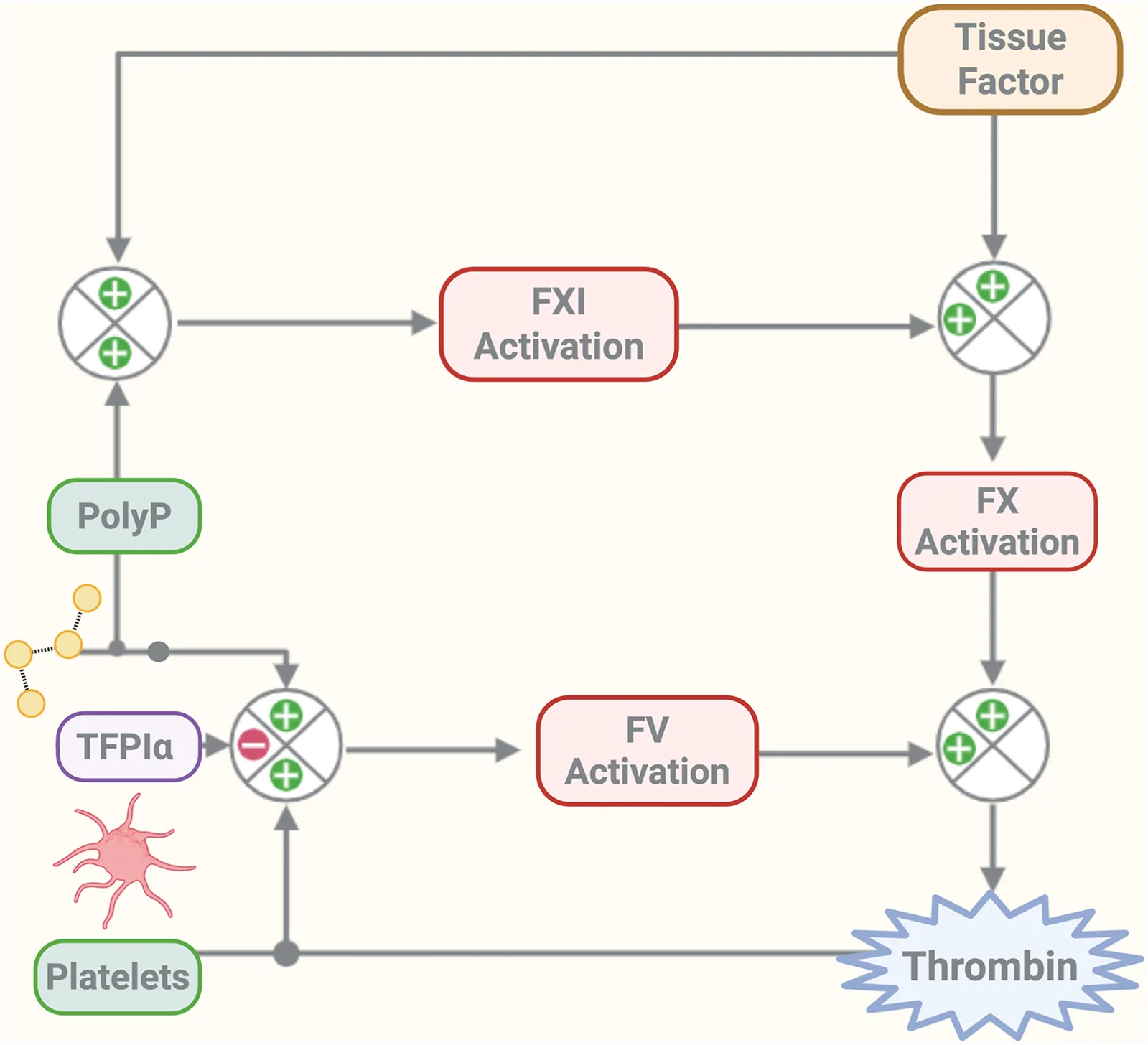
Block diagram of thrombin generation. Tissue factor–initiated coagulation catalyzes platelet activity and release of short-chain polyP and tissue factor pathway inhibitor α (TFPIα), which sum to regulate FVa and subsequent thrombin generation.

Under accelerating shear flow, convection of cofactors and unfolding of von Willebrand factor, a shear-responsive glycoprotein, activates platelets to produce a phosphatidylserine-rich surface which potently catalyzes thrombin generation [3]. Consequently, the downstream activation of FXI by thrombin induces a positive feedback loop to amplify thrombin generation [4]. Moreover, activated platelet surfaces express and release mediators stored in their granules including adenosine diphosphate, P-selectin, and short-chain polyphosphate (polyP), which coalesce to amplify and sustain platelet activation and thrombin generation [5].

Platelet-derived short-chain polyP, an inorganic polymer of phosphate ions, serves as a cofactor which accelerates the kinetics of FXI and FV activation to amplify thrombin generation [5]. In contrast to the catalytic function of short-chain polyP, activated platelets also secrete TF pathway inhibitor α (TFPIα), a serine protease inhibitor which binds the B-domain of FV and binds FXa to inhibit downstream cleavage of prothrombin to thrombin [2]. Notably, short-chain polyP has been demonstrated to reduce the anticoagulant activity of TFPIα. By accelerating the activation of FV and FXI (FXIa), polyP attenuates the rate-limiting effect of TFPIα on thrombin generation and, consequently, pharmacologic inhibition of platelet-derived polyP has been shown to enhance the ability of TFPIα to suppress procoagulant activity [6]. By leveraging computational models, these complex and intradependent causal loops can be mapped to predict the relative contributions of catalytic surfaces and cofactors in the generation or inhibition of thrombin, all within the context of dynamical flow conditions [7,8].

In this issue of *Research and Practice in Thrombosis and Haemostasis*, Ramesh Bhatt et al. [9] advance an established mathematical model of TF-initiated coagulation under flow to include the kinetic contribution of platelet-derived short-chain polyP on thrombin generation in the plasma boundary layer. Building upon the work of several studies [[10], [11], [12], [13], [14]], the investigation herein models a control volume of peripheral plasma flow across a site of exposed extracellular matrix. The authors develop an “implicit” steady-state model to predict the effects of platelet-derived polyP on thrombin generation by varying the catalytic rate constants of FXI and FV activation reactions in the absence or presence of polyP. In addition, an “explicit” model is extended to deduce how these rates vary with respect to the relative diffusion and residence times of platelet-bound polyP (P^b^) and fluid-phase polyP (P^f^) as they are recruited to activated platelet surfaces and removed via flow.

The results show that inclusion of platelet-derived polyP within the implicit model provoked rapid thrombin generation (the “thrombin burst”) across all TF densities under low shear rates yet primarily demonstrated increased thrombin concentration at 10 minutes ([Thrombin]_10min_) when TF density was lowered. Moreover, the sensitivity of thrombin generation to physiological shear across rates of 100 to 1500 s^−1^ was substantially reduced in the presence of platelet-derived short-chain polyP. Upon TF densities exceeding ∼0.3 fmol/cm^2^, the role of platelet-derived polyP in thrombin generation is shifted toward FV rather than FXI. An enhanced role for partially activated FV (FVh) was observed for conditions where FXI activation was accelerated by platelet-derived short-chain polyP, whereas the sensitivity of thrombin generation to TFPIα was abrogated in a TF-dependent manner.

Analysis of the explicit model presented 3 variables which represent dynamic parameters of FXI and FV activation by short-chain polyP released from platelets and captured on activated platelet surfaces. Increased number of platelet binding sites (n_bs_), tighter theoretical binding of short-chain polyP to activated platelet surfaces (K_D_), and increased sensitivity of catalytic rates (k∗) to P^b^ concentration were predicted to accelerate thrombin generation by short-chain polyP at TF densities below ∼2.6 fmol/cm^2^. These parameters were summarized in the ratio R = (n_bs_/K_D_)/k∗, which describes the respective time dependencies of P^b^, P^f^, and thrombin.

This study advances the mathematical modeling of thrombin generation under flow through novel computational data of platelet-released polyP function and furthers the utility of computational simulation to investigate coagulation kinetics. Future refinements to the model may include addition of shear-induced kinetics of thrombin as a species in contrast to representing thrombin concentration as a purely time-dependent covariate. Furthermore, dynamic similarity of some referenced literature values could be reevaluated with respect to the explicit model. Other contributing biological factors which may influence thrombin generation include inactivation of TFPIα by FXIa [15] and potential interactions between platelet factor 4 and platelet-released polyP [16], in addition to consideration of complete vessel occlusion and pulsatile flow in select vascular beds in the explicit model.

Altogether, the mathematical predictions offered by Ramesh Bhatt *et al.* present a compelling narrative of the effects of platelet-released polyP on thrombin production under flow conditions in TF-initiated coagulation. Although the calculated rates of thrombin generation remain to be validated in assays including organ-on-chip and *in vivo* models, the findings from this study call for future evaluation of the translational potential for targeting polyP as a means to reduce pathologic thrombin generation while preserving hemostatic function [17,18]. Most significantly, the mathematical system presented by the authors strengthens the functionality of computational modeling as a preliminary means to investigate platelet and coagulation factor kinetics under simulated physiological flow.
